# Resilience and vulnerability among refugee children of traumatized and non-traumatized parents

**DOI:** 10.1186/1753-2000-2-7

**Published:** 2008-03-28

**Authors:** Atia Daud, Britt af Klinteberg, Per-Anders Rydelius

**Affiliations:** 1 Karolinska Institutet, Dept. of Woman and Child Health, Child and Adolescent Psychiatric Unit, Astrid Lindgren's Children's Hospital, Stockholm, Sweden; 2 Stockholm University, Dept. of Psychology, section for Biological psychology, Stockholm, Sweden; 3 Stockholm University/Karolinska Institutet, Centre for Health Equity Studies, Stockholm, Sweden

## Abstract

**Background:**

The aim of the study was to explore resilience among refugee children whose parents had been traumatized and were suffering from Post-Traumatic Stress Disorder (PTSD).

**Methods:**

The study comprised 80 refugee children (40 boys and 40 girls, age range 6–17 yrs), divided into two groups. The test group consisted of 40 refugee children whose parents had been tortured in Iraq before coming to Sweden. In accordance with DSM-IV criteria, these children were further divided in two sub-groups, those who were assessed as having PTSD-related symptoms (n = 31) and those who did not have PTSD-related symptoms (n = 9). The comparison group consisted of 40 children from Egypt, Syria and Morocco whose parents had not been tortured. Wechsler Intelligence Scale for Children, 3^rd ^edn. (WISC-III), Diagnostic Interview for Children and Adolescents- Revised (DICA-R), Post-Traumatic Stress Symptoms checklist (PTSS), "I Think I am" (ITIA) and Strengths and Difficulties Questionnaire (SDQ) were used to assess IQ; PTSD-related symptoms; self-esteem; possible resilience and vulnerability.

**Results:**

Children without PTSD/PTSS in the traumatized parents group had more favorable values (ITIA and SDQ) with respect to *total scores, emotionality*, *relation to family*, *peer relations *and *prosocial behavior *than the children in the same group with PTSD/PTSS and these values were similar to those the children in the comparison group (the non-traumatized parents group). The children in the non-traumatized parents group scored significantly higher on the IQ test than the children with traumatized parents, both the children with PTSD-related symptoms and those without PTSD-related symptoms.

**Conclusion:**

Adequate emotional expression, supportive family relations, good peer relations, and prosociality constituted the main indicators of resilience. Further investigation is needed to explore the possible effects of these factors and the effects of IQ. The findings of this study are useful for treatment design in a holistic perspective, especially in planning the treatment for refugee children, adolescents and their families.

## Introduction and theoretical basis of the study

Children in families that have suffered trauma constitute a risk group for developing psychiatric illness, dysfunctional behavior and inadequate academic achievement. Child maladaptive stress syndrome has been shown to be associated with parental psychiatric illness [1].

The association between parental trauma and children's symptomatology has been explored among children of Holocaust survivors [2]. Findings of the Holocaust study indicated that there is a relationship between parental trauma and their children's PTSD symptoms, which gives support to the hypothesis of a transgenerational transmission of trauma impact. Research has shown the importance of including the family's history of psychopathology as environmentally mediated psychosocial risk factors and as determinants in the child's development of cognitive/affective internal working models [3-5]. Otto and associates [6] investigated the association between PTSD symptoms and children's television viewing in the USA. One of their findings was that some 5% of the children who had seen the 9/11 tragedy on television developed symptoms of PTSD. This finding supports the hypothesis of indirect traumatization in children.

A recent study has shown that, as a response to life stressors, such as exposure to violence or a death in the family, adolescents may develop internalizing symptoms such as depression, PTSD, and anxiety and/or externalized symptoms such as substance use, aggression, and delinquency [7]

## Resilience and protective factors vs. vulnerability and risk factors

Resilience in children has been operationalised in various ways. Garmezy et al. [8] defined resilience as the manifestation of competence in children although they have been exposed to stressful events. Gordon [9] emphasized the individual's capacity to thrive, mature, and develop competence despite adverse circumstances. For Crawford and associates [10], resilience means that the individual has the ability to adapt under stress, particularly in the context of severe hardship and disadvantageous life circumstances. Garmezy [11] formulated three factors that in particular promote the development of resilience in children: 1) the child's personality dispositions; 2) a supportive family environment; and 3) a support system outside the family that encourages and reinforces the child's efforts to cope and instills in the child positive values.

Close affirmative relationships, continuous and personalized care-giving, appropriate teaching and learning experiences, and an external social group with a supportive ethos and behavioral styles are all factors that protect the child from developing maladaptive behavioral patterns [12].

Resilience and protective factors have also been conceptualized [13] as the antithesis to vulnerability and risk factors. In the context of the present study, vulnerability means heightened susceptibility to develop PTSD/PTSS or a clinical picture dominated by PTSD-related symptoms. The risk factors that can lead to a negative developmental outcome include emotionally stressful relations in the family, the lack of continuity in care-giving, the lack of appropriate teaching and learning experiences, and participation in a social group with a deviant ethos or behavioral styles [11]. An example of research in this field of inquiry is Rydelius' ([3,4] longitudinal study of psychosocial risk factors among a group of children with alcoholic fathers. This research accentuated the significance of psychosocial stress factors in the development of psychopathology [15].

Although it is important to identify risk factors, it is equally important to identify the protective factors that are present in the family, peer group, and school environment. In a study of protective factors within the family, Rutter [16] showed that psychosocial investigations of families need to include the family's past experiences as well as their current life circumstances.

Resilience has also been conceptualized as a universal human capacity to cope with traumatic events, but that this capacity needs encouragement and support within a facilitative environment to enable resilience to win over vulnerability and risk [14]. This conceptualization has shifted the focus away from individual deficits to individual strengths, competencies, and capacities and was a critical step in understanding resilience within the context of the individual and the family [14].

Resilient children are socially competent, have a positive self-esteem, and a sense of their own efficacy and ability. They possess above average intelligence expressed in terms of IQ, which may enhance their coping strategies, and they are able to understand and express a wide range of emotions in a socially appropriate manner. [17,18].

In summary, resiliency has been regarded as the individual's capacity to adapt in the face of threatening circumstances and to develop strategies to cope with conditions of prolonged or severe adversity [13]. Werner and Smith [13] suggested that this works through the interaction of factors comprising: (i) dispositional attributes of the child, such as intelligence, sociability, effective coping strategies, and communication skills; (ii) family characteristics such as warm relationships, cohesion, structure, emotional support, secure attachment, and a close bond to at least one caregiver; and (iii) external support factors such as positive school experiences, good peer relations, and positive relations with other adults.

## Personality-related aspects and PTSD

A closely related concept is hardiness. Kobasa [19] defined hardiness as a constellation of personality traits that facilitates the development of strategies for coping with stressful life events, also as an adaptive attribute based on early learned social cognition and characterized by rich and varied experiences. However, Kobasa [19] expanded the definition of hardiness to encompass *commitment*, a sense of meaning, purpose, and perseverance associated with one's existence; *control*, a sense of autonomy, endurance, and the ability to influence one's own life course; and *challenge*, the perception of change as a normal aspect of life and as an opportunity for growth (see also [20]). Bartone [21] investigated the relationship between hardiness, combat exposure, and PTSD symptoms in veterans of the Gulf War and found that the 'hardy' veterans displayed fewer PTSD symptoms. The risk of developing PTSD among the Gulf War veterans was aggravated by such factors as family instability, poor family relations in general, as well as their particular war-zone experiences [22]. Social support, i.e. the interpersonal resources that promote hardiness, was a resilience factor. Another important factor was the availability of sources of support in the environment, which further enhanced the individual's possibility to develop hardiness, decreased the likelihood of developing PTSD symptomatology, and created opportunities for developing resilience.

## Research questions and the aim of the study

The occurrence and absence of trauma-related psychopathology, primarily PTSD or PTSS, in the children of traumatized parents [1] raised the question of which dispositional features of the child's personality and what environmental factors were at work to explain why some children did not show PTSD-related symptoms.

The aim of the present study was to explore resiliency among children who did not develop PTSD-related symptoms despite a history of parental PTSD.

In the present study, prosocial behavior and psychological wellbeing are hypothesized as reflecting person-related attributes, which can strengthen a good to *relation to family*, which in turn functions as an environmental protective factor. An adequate self-esteem in the present study, measured by the ITIA with results according to Stanine -Scale above 5 Stanine, is assumed to facilitate the person-related components in the concept of resilience.

Three hypotheses were formulated: (i) that *self-esteem*, including *prosocial behavior, psychological wellbeing*, and *IQ *were factors that facilitated resilience; (ii) that adequate *relation to family*, measured by ITIA, was a protective factor; and (iii) that resilient children in the traumatized parents group, i.e. children without PTSD-related symptoms, will have higher scores on the SDQ regarding *emotionality variable *and on the *peer problems variable *than the children in the same group with PTSD-related symptoms.

## Methods

### Participants

#### The parents and their children

The test group (the traumatized parents group) consisted of 15 refugee families (30 parents) from Iraq (mean age 41.1), with documented torture experiences. In all but one family, both fathers and mothers had experienced torture. They were selected in accordance with the project's three inclusion criteria: (i) being subjected to severe torture for duration of at least one month; (ii) having children between 6–17 years of age; (iii) living in Sweden for at least two year before participating in the study. The participating families were recruited from the Swedish Red Cross' Centre for Tortured Refugees and the Centre for Trauma Treatment and Diagnostics in Stockholm (CTD) where they had, or were currently, receiving psychiatric/psychotherapeutic treatment.

The traumatized parents' "Torture experiences" was used as a concept that included such aspects as forced separation from the family, near-death experiences, imprisonment, and torture. We did not differentiate between the varieties of torture acts or their outcome in terms of developing or not developing PTSD. We used the torture concept as a cumulative matrix of traumatic events which may have caused PTSD.

The comparison group (the non-traumatized parents group) consisted of 15 refugees families from Egypt, Syria and Morocco (26 parents with mean age 42.2) who had no self-reported experiences of torture or violence prior to coming to Sweden. In four of the families, due to divorce, there was only one parent living with the children, which reduced the number of parents in the study to 26. The 15 families who participated in this group were recruited from immigrant associations in the greater Stockholm area and all of them replied affirmatively to our letter of invitation to participate. All 30 families in the two groups had come to Sweden during the former regime in Iraq and before the ongoing Iraqi war.

The educational level of the parents in both groups was compared. Fourteen of the 30 parents in the test group had a senior high school, college, or university education compared with 19 of the 26 parents in the comparison group.

The families had been investigated in an earlier study to explore possible transgenerational transmission of parents' traumatic experiences to their children [1]. At the time of that study, there were 45 children in the traumatized parents group and 31 children in the non-traumatized parents group. All the children were between the ages of 6 and 17, which was the age range for inclusion in the earlier study. For the present study, the age range for inclusion was somewhat more narrow, 7–16 years. In the traumatized parents group the oldest children from the earlier study now exceeded the 6–17 years age range, which thereby excluded them from the study, while a few younger children (born in Sweden) now entered the age range. In all, this reduced the number of children in this group from 45 to 40. In the non-traumatized parents group, a similar situation change occurred. However, here younger children (born in Sweden) entered the age range for inclusion, which increased the number of children in this group from 31 to 40. The two groups of parents consisted of the same individuals in both studies.

In summary, in the present study, the total sample was 80 children, 40 girls and 40 boys, aged 7–16 years; all were of Arabic ethnicity and language. Forty of the children (n = 40, mean age 12.1, SD 2.1) belonged to the traumatized parents group and 40 (n = 40, mean age 12.5 and SD 2.2) belonged to the non-traumatized parents group. All the children in the present study were born in Sweden.

The children's inclusions criteria were: (i) refugee children of traumatized/non-traumatised parents; (ii) age between 7–16 years; and (iii) Arabic ethnicity, Arabic language; and (iv) enrolled in the regular Swedish school system

## Instruments and measures

In all, five instruments were used in the study:

- The revised version of the *Diagnostic Interview for Children and Adolescents *(DICA-R). This semi-structured clinical interview schedule was used to assess the presence of PTSD-related symptoms among the 80 children in the sample.

- Children's self-rating on the Post-Traumatic Stress Symptoms checklist [23,24].

- The *Wechsler Intelligence Scales for Children*, Third edition (WISC-III). The raw scores of the WISC-III measured the IQs of all 80 children with respect to VIQ (Verbal IQ), PIQ (Performance IQ), and FSIQ (Full scale IQ).

- The *'I think I Am' *(ITIA) *Questionnaire*, which is also a self-report instrument for the purpose of measuring children's self-esteem.

- Teacher ratings according to the Strengths and Difficulties Questionnaire (SDQ) [24] were used to assess children's emotional symptoms, behavioral problems, hyperactivity, and peer problems.

In summary, the children of these families were examined concerning (i) self-esteem and (ii) IQ as main factors that may influence resilience. The children's vulnerability, operationalized in terms of developing PTSD-related symptoms was examined. The children in the traumatized parents group were divided into those with PTSD-related symptoms and those without PTSD-related symptoms. Self-esteem was assessed using the children's self-reports on the *'I Think I Am' (ITIA) Questionnaire*. IQ was measured using the WISC-III. Vulnerability, expressed as PTSD-related symptoms, was measured using the Diagnostic Interview for Children and Adolescents Revised (DICA-R) and the children's self-ratings on the Post-Traumatic Stress Symptoms checklist.

For readers outside Scandinavia, it might be appropriate to describe the ITIA instrument in more detail. The 'I think I Am' (ITIA) Questionnaire' for measuring self-esteem is a Swedish self-report scale developed and standardized on a sample of over 3,465 children between 8–16 years of age [25]. It consists of 72 items divided into five factors that measure the child's ideation about him-/herself with respect to: *physical components, skills and talents, psychological wellbeing, relation to family, relation to others; *and lastly, the child's *total score*. The child is asked to choose from among four alternatives: *'Exactly like me', 'Almost like me', 'Not quite like me' *and *'Not at all like me'*. The ITIA total composite score ranges between +144 and -144. A high score on the ITIA questionnaire indicates that the child has adequate mental health.

The theoretical basis of the ITIA questionnaire rests on the work that has been done on the concept of self-esteem [27], on measures of children's self-concept [27], and on measures of children's self-image [28]. The validity and reliability of the ITIA have been extensively investigated [29], as the ITIA is widely used in clinical settings in Sweden to investigate the psychological wellbeing of children suffering from somatic illness [30-32].

The Strengths and Difficulties Questionnaire (SDQ) [25] is available in a Swedish version. Teacher ratings of the established SDQ scales were used to assess children's resiliency/vulnerability: *emotional symptoms, behavioral problems, hyperactivity, peer problems, prosociality*, and *total difficulty*. In accordance with the on-line Swedish SDQ instructions, the following categorization was used:*"Normality," "Borderline," *and *"Abnormality."*

### PTSD diagnostic features in children

The DSM-IV TR [33] outlines the essential criteria for ascertaining PTSD following exposure to extreme traumatic stressors involving direct personal experiences of the threat of death or serious injury or other threat to the individual's physical integrity, or the witnessing of these threats against another person, or hearing about an unexpected or violent death of a family member or other close associate, or that the family member or other close associate has suffered serious harm or the threat of death or injury (Criterion A1). It has been shown [34] that hearing about one's parents' traumatic experiences may in itself be a contributing cause of the child's developing partial PTSD. In the present study, although there was no indication that the children themselves had experienced traumatic events, there is evidence that they knew about their parents' torture experiences as something very horrifying. In accordance to the PTSS checklist [23,24] used in the study and to assess for Criterion A1, each child in our study was asked the following question: *What is the most horrifying event you have experienced heard about*. The responses from all 40 children in the traumatized parents group were of the kind: "It was when my father was in jail under Saddam;" "When my father was captured and my mother didn't know where he was;" and "When my father was in jail and my uncle was executed and my mother was afraid everyone would be executed." To assess for Criterion A2 ("The person's response involved intense fear, helplessness, or horror; Note: In children, this may be expressed instead by disorganized or agitated behavior), the Diagnostic Interview for Children and Adolescents (DICA) was used together with the PTSS Checklist [23,24]. The PTSS checklist includes post traumatic stress symptoms in according to the DSM-classification in three symptom clusters: Re-experiencing the event (4 items), Avoidance of reminders and emotional numbness (7 items) and Hyperarousal (6 items). The results from the open interviews and the answers to the questionnaires showed that the children's answers on the PTSD/PTSS items were all related to the parent's torture experiences which in retrospect were used to assess A2.

## Procedure

The parents in both groups had been assessed in an earlier study [1] regarding PTSD using a semi-structured clinical interview administered by a psychiatrist, and the H/UTQ administrated by the first author (A.D.). This was done to re-assess the psychiatric status of the parents in the traumatized group who had had clinical treatment and to assess the non-traumatized parents for PTSD/PTSS. The parents were also investigated using the *Karolinska Scales of Personality *(KSP). However, no IQ tests were performed on the parents. Because there were no histories of new parental traumatic experiences since the earlier study, no re-assessment of the parents was made for the present study.

In summary, the children's assessment for PTSD-related symptoms was made by a psychiatrist and the IQ and ITIA tests were conducted by a clinical psychologist. SDQ ratings were made by the teachers and were computed by clinicians, and, finally, the KSP questionnaires used in the earlier study were conducted by a clinical psychologist.

The study design included two steps. In Step1, all the children were first assessed using the five instruments/questionnaires described above. The possible presence of PTSD-related symptoms among the 80 children was assessed by means of the *Diagnostic Interview for Children and Adolescents *(DICA-R) and the children's self-rating on PTSS checklist. The cut-off score for posttraumatic symptoms according to DICA is > than five symptoms, besides the answer *yes *to the question of whether they had heard about horrifying events affecting their family. The rationale for using the cut-off procedure with more than five symptoms was based on the DSM-IV TR. system.

All the children were investigated using both the children's native language and the Swedish language. There was nothing in the histories of either group of children to indicate that any of them had personally experienced traumatic events such as torture, parental abuse, or domestic violence.

The raw scores of the WISC-III were used to measure VIQ (Verbal IQ), PIQ (Performance IQ), and FSIQ (Full-Scale IQ) among the whole sample. The self-rated ITIA and the WISC-III tests took three hours per child to complete and were conducted at the children's respective schools.

The children completed the ITIA questionnaire individually and their teachers, who were not informed of the purpose of the study, rated the SDQ for each child. This procedure was completed in the middle of the academic year to give the teachers time to form an objective opinion of the child.

High scores (> 5 "Stanine Scale") on the ITIA sub-scales *psychological wellbeing*, *relation to family*, and *relation to others*, together with scoring < the abnormal level on the SDQ sub-scales *prosocial behavior and peer relations *are conceptualized as environmentally moderated components that enhance the development of resilience and are assumed to reflect protective factors characterized by supportive family relationships and external social support.

Resilience was operationalized and defined in the study as the child's high scores on the 'I think I Am' (ITIA) questionnaire and as the child's low score [31] on the sub-scales of the Strengths and Difficulties Questionnaire (SDQ) apart from the sub-scale '*prosocial behavior,' *on which a high score is positive.

Step 2. Based on the results from the DICA-interviews, the children in each parent group were to be divided into two sub-groups, those children with PTSD-related symptoms and those children without PTSD-related symptoms. The results from the DICA-interviews showed, however, that none of the children in the non-traumatized parents group had PTSD/PTSS. Therefore, for the further analysis the children were divided into three groups:

1) Children in the traumatized parents group with PTSD-related symptoms;

2) Children in the traumatized parents group without PTSD-related symptoms;

3) Children in the non-traumatized parents group (comparison group)

## Treatment of data and statistical analysis

For significance testing of equality of means, the Student's *t*-test was used. The chosen significance level was 0.05. F-ratio for one-way ANOVA and significance (Tukey, 1%) for sub-group comparisons were computed. The Pearson correlation coefficients were used to estimate the association between PTSD symptoms and IQ variables, and to estimate the association between ITIA and SDQ variables. Significance level of 5% was chosen. The calculations were made using SPSS, version 11.0.

## Ethical considerations

The local ethical committee at Karolinska Hospital in Stockholm approved the study

(Dnr 97–295, 2000-06-05). All parents were informed about the purpose of the research project and that their identities would be kept anonymous throughout the whole data processing and presentation of the findings. All the participating subjects gave their informed consent and their participation was wholly voluntary.

## Results

### PTSD in parents' and PTSD related symptoms in children

The parents in the whole sample (test and comparison groups) were assessed concerning PTSD. All the parents in the traumatized group, 14 mothers and 15 fathers had been assessed as having PTSD while none of the parents in the non-traumatized group had PTSD/PTSS [1].

Among the children in the traumatized parents group, 31 Ss (17 boys, mean age 12.5 years, S.D 2.0; and 14 girls, mean age 12.8, S.D. 2.5) showed PTSD-related symptoms according to DICA-R, while the remaining 9 Ss (3 boys and 6 girls) did not. Among the children in the non-traumatized parents group, no-one showed PTSD-related symptoms. A comparison by age between the children in the traumatized parents group with PTSD-related symptoms and those without PTSD-related symptoms was non-significant (*t *= 1.52, *p *= ns).

As described in the section on Methods and Procedure, for the further analysis the children were then divided into three groups: 1) children in the traumatized parents group with PTSD-related symptoms; 2) children in the traumatized parents group without PTSD-related symptoms; and 3) children in the non-traumatized parents group (comparison group).

### Intelligence (IQ) and PTSD-related symptoms in both groups of children

Pearson correlations for the whole sample between the number of PTSD-related symptoms and IQ variables showed significant negative correlations: VIQ (r = -.52; p < 0.001), PIQ (r = -.44; p < 0.001), FSIQ (r = -.52; p < 0.001). The children in the non-traumatized parents group had statistically significantly higher scores for VIQ, PIQ, and FSIQ than the children in the traumatized parents group, including both those with and those without PTSD/PTSS (p < 0.01). Among the children in the traumatized parents group, those not showing PTSD-related symptoms had on average a VIQ of 91.7 vs. 86.7 in the group showing PTSD-related symptoms. See Table 1.

**Table 1:** Mean scores (M) and standard deviations (SD) on Verbal IQ (VIQ), Performance IQ (PIQ) and Full Scale IQ (FSIQ) in a group of children (n= 80) divided into three subgroups: children in the traumatized parents group without PTSD-related symptoms (n = 9), children in the traumatized parents group with PTSD-related symptoms (n = 31), and children in the non-traumatized parents (n = 40). F-ratio for one-way ANOVAs (*df2.78*) and significance (Tukey 1%) for subgroup comparisons

IQ	Traumatized Non-PTSD-related symptoms **A**		Traumatized PTSD-related symptoms **B**		Non-Traumatized **C**				*Post-hoc *test p < 0.01
		
	*M*	SD	*M*	SD	*M*	SD	*F*	*p*	
VIQ	91.7	14.3	86.7	11.1	104.2	12.4	18.8	<.001	C > A, B
PIQ	83.4	16.5	86.2	13.9	100.7	14.8	10.7	<.001	C > A, B
FSIQ	86.4	15.9	86.5	12.1	102.5	13.2	18.3	<.001	C > A, B

*Note*: **A **= Children without PTSD-related symptoms in the traumatized parents group;

**B **= Children with PTSD-related symptoms in the traumatized parents group;

**C **= Children in the non-traumatized parents group.

### Self-esteem according to ITIA and PTSD

The children from the non-traumatized families had higher scores regarding the ITIA *psychological wellbeing *(p < 0.05) and *total score *(p < 0.05), and a tendency to show better *relation to family *(p = 0.06) compared with the children from the traumatized families. When comparing the three sub-groups of children, those with PTSD-related symptoms and those without PTSD-related symptoms, no significant differences were found. However, children without PTSD-related symptoms had, irrespective of family background, more similar values on the sub-scale *relation to family *and total score than the children showing PTSD-related symptoms. The children not showing PTSD-related symptoms from the traumatized families had the highest scoring on *relation to others*. See Table 2.

**Table 2:** Mean raw scores (M) and standard deviations (SD) of ITIA conceptualized as resilience factor among children (n= 80) divided into subgroups with children in the traumatized parents group without PTSD-related symptoms (n = 9), children in the traumatized parents with PTSD-related symptoms (n = 31), and children of non-traumatized parents (n = 40).

ITIA variables	Traumatized Non-PTSD- related symptom **A**		Traumatized PTSD-related symptoms **B**		Non-Traumatized **C**				*Post- hoc *test p < 0.01
		
	*M*	*SD*	*M*	*SD*	*M*	*SD*	*F*	*p*	
Psychological wellbeing	13.8	7.9	9.0	8.1	15.1	7.8	1.1	0.05	C > B
Physical components	16.2	6.8	16.4	6.8	17.1	7.4	0.6	n. s	
Relation to family	16.5	6.5	17.9	7.1	20.0	7.4	2.4	n. s	
Relation to others	13.5	5.5	9.9	6.6	13.8	6.8	0.9	n. s	
ITIA Total score	69.8	29.7	59.9	31.4	80.2	30.8	1.4	0.05	C > B

Note*: **A **= Children without PTSD-related symptoms in the traumatized parents group;

**B **= Children with PTSD-related symptoms in the traumatized parents group;

**C **= Children in the non-traumatized parents group.

Note**: The ITIA's sub-scale "Relation to family" C > A+B, t-value 1.9; p =< 0.06 also the ITIA's "Total score" C > A+B, t-value 2.1; p < 0.05

### SDQ scores and PTSD

Children without PTSD/PTSS, irrespective of family background, had more positive scores on the SDQ sub-scales (p < 0.001). Relatively, as shown in Table 3, children without PTSD-related symptoms from traumatized families had the lowest scores on the *emotionality*; *hyperactivity*, and *peer problems *sub-scales and the highest scores on the sub-scale *prosocial behavior *indicating both good competence and behavior. See Table 3.

**Table 3:** Mean raw scores (M) and standard deviations (SD) of SDQ among refugee children of traumatized parents without PTSD-related symptoms; refugee children of traumatized parents with PTSD-related symptoms and refugee children of non-traumatized parents denoted as Non-traumatized without PTSD-related symptoms group (n= 80).

SDQ variables	Traumatized without PTSD-related symptoms **A**		Traumatized with PTSD- related symptoms **B**		Non- traumatized without PTSD-related symptoms **C**				*Post-hoc *test p < 0.01
		
	*M*	SD	*M*	SD	*M*	SD	*F*	*p*	
Emotionality	4.2	2.0	5.7	2.3	2.2	2.0	15.3	< 0.001	C < A, B
Hyperactivity	5.2	2.9	6.6	2.8	2.6	2.0	15.2	< 0.01	C < A, B
Peer problems	3.9	2.0	4.6	2.5	2.7	2.2	4.2	< 0.05	C < B
Prosocial behaviour	6.1	3.2	6.1	3.6	7.6	1.8	3.4	n. s.	
SDQ Total Score	16.6	7.7	20.6	7.7	9.1	6.1	19.0	< 0.001	C < B, A

*Note*:**A **= Children without PTSD-related symptoms in the traumatized parents group;

**B **= Children with PTSD-related symptoms in the traumatized parents group;

**C **= Children in the non-traumatized parents group.

### Self-esteem according to ITIA, SDQ and PTSD-related symptoms

The Pearson correlations between self-esteem (the ITIA total score) and SDQ variables for the whole sample showed a significant negative correlation between high self-esteem and low scoring (no problems) on the SDQ's *emotionality *scale (r = -.31; p < 0.01).

### A comparison between those children in the traumatized families with PTSD-related symptoms and those children without PTS- related symptoms

Significant differences with respect to resilience and protective factors and in favor of the children not showing PTSD-related symptoms was found when comparing those without, as follows: *emotionality *(p < 0.01), *peer problems *(p < 0.001), *prosocial behavior *(p < 0.05), and *total score *(p < 0.001). Furthermore, the children without PTSD-related symptoms tended to have higher scores on the sub-scales *psychological wellbeing *(p < 0.05), *total score *(p < 0.05), and *relation to family *(p < 0.06). See Table 4.

**Table 4:** Mean raw scores (M) and standard deviations (SD) of resilience according to ITIA and SDQ variables in children with PTSD-related symptoms (n = 31) and without PTSD-related symptoms (n = 9) in the traumatized parents group.

Resilience according to ITIA and SDQ variables	Children of traumatized parents					
			
	PTSD-related symptoms		Non-PTSD- related symptoms		*t*-values	*p*
			
	M	SD	M	SD		
ITIA Relation to family	16.8	7.1	20.0	7.4	1.9	< 0.06
ITIA Total score	69.8	29.7	80.2	30.8	1.4	< 0.05
SDQ Prosocial behavior	6.4	3.1	8.0	1.7	2.1	< 0.05
SDQ Emotionality	4.4	2.1	1.6	1.6	4.2	< 0.01
SDQ Peer problems	3.7	1.9	1.1	1.2	5.0	< 0.001
SDQ Total Impairment score	16.7	6.3	6.0	4.2	5.9	< 0.001

*Note*: PTSD-related symptoms = Children with PTSD-related symptoms in the traumatized parents group; and Non-PTSD-related symptoms = Children without PTSD-related symptoms in the traumatized parents group.

## Discussion

Children of traumatized parents as an overloaded group, especially with respect to their susceptibility to developing psychiatric disorders (mainly PTSD- or PTSD-related symptoms), were the target of an earlier investigation [1]. Living with a traumatized parent is in itself a very severe and threatening circumstance. The fear of losing the parent; the fear that the parent will re-experience the life-threatening event again even in the new country; the question of whether the parent's capacity to be '*good enough parent*' is insufficient; all this threatens the fundamental secure base which is needed for the child's adequate psychological development in terms of secure attachment.

It was noted in the earlier study, however, that some of the children did not develop PTSD/PTSS. As was also found in the Ferren study [36], these children displayed salutogenic features (freedom from PTSD/PTSS) as a consequence of their resilience which was characterized by their maintaining adequate family and peer relations.

They also displayed adequate emotionality and had a low score on the total impairment measure. The children without PTSD-related symptoms in the traumatized parents group might have hardiness as main construct in the concept of protective factors enhancing these salutogenic outcomes. Goldstein and Brooks [43], in their *Handbook of Resilience in Childhood *from 2006, wrote: "Resilience is suggested as but one of a number of constructs that protect or reduce vulnerability. Lösel, Bliesener, and Köfrel (1998) suggested that other protective factors include hardiness, adaption, adjustment, mastery, good fit between the child and the environment and buffering of the environment by important adults in the child's life" (p. 5).

The findings in the present study also support the hypothesis that there is a relationship between high self-esteem as a main factor in resilience and the development of salutogenic features. Using self-esteem (prosocial behavior together with psychological wellbeing) as an indicator of resilience and the SDQ sub-scale *peer relations *as an environmental protective factor enabled us to investigate the quality of the children's relationship to their significant others according to the ITIA scales. Another finding indicates that the child's adequate relation to his/her family (the supportive family) promoted the development of salutogenic features, even in the face of the parents' own lack of wellbeing.

Children in the traumatized parents group who displayed PTSD/PTSS scored significantly lower on the *prosocial behavior *scale – i.e. the child's capacity to manage relations with others and to be helpful – than did the children in the same group without PTSD-related symptoms. It is likely that there is a relationship between these children's low scores on the *prosocial behavior *scale and their parents' symptomatology, but much unexplained variability remains. Some of the children in this group did not develop these dysfunctional behaviors. This is in line with results obtained in a study of male subjects with a history of childhood victimization [37]. The overall findings of the present study support the hypothesis that the presence of environmental protective factors which facilitate the children's social competence may have enhanced the development of a functional salutogenic/protective mechanism and resilience factors among children in the traumatized parents group without PTSD-related symptoms. The parents' lack of wellbeing does not seem to have affected these children's behavior.

Although the concept of salutogenesis as formulated by Antonovsky was not used in this study, the results of the psychological and physical components of the ITIA sub-scales were not significant despite their encompassing what Antonovsky denoted as the biological domain of salutogenesis. On the other hand, the results obtained on the SDQ sub-scales *emotionality *and *prosocial behavior *also qualified as personality attributes similar to what has been termed the psychological domain in salutogenesis [38,39]. The term salutogenesis [40-42] highlights the aspects of wellbeing rather than of pathogenesis. Antonovsky observed that some Holocaust survivors had fared relatively well despite their overwhelming negative experiences. He proposed that the core factor in salutogenesis is the individual's possession of a high sense of coherence (SOC). SOC is defined as 'a global orientation that expresses the extent to which one has a pervasive, enduring and dynamic feeling of confidence; that one's internal and external environments are predictable and that there is a high probability that things will work out as well as can be reasonably expected' [8]. SOC, which is comprised of comprehensibility, manageability, and meaningfulness, is an essential component of the individual's sense of wellbeing.

Antonovsky's interpersonal domain may be operationalised as the ITIA sub-scale *relation to family *(p < 0.06) and the SDQ's sub-scale *peer problems *(p < 0.001), as studied among children of traumatized parents. If so, the findings indicate that having a supportive family and adequate relations to others are main factors in understanding both protective factors and salutogenesis. To reiterate, further research is needed to explore the complexities of the salutogenic framework of those children in the present study who, although their parents were traumatized, did not develop PTSD-related symptoms.

The children in the non-traumatized parents group had significantly higher scores on the WISC-III with respect to VIQ, PIQ and FSIQ compared with the children in the traumatized parents group. As there was some difference referring to the educational level of the two parental groups, and as no IQ-tests were performed when investigating the parents, this result is not surprising. Although the children from the traumatized parents not showing PTSD-related symptoms showed relatively higher values on the VIQ, our results did not support the hypothesis that IQ was a factor involved in understanding resilience in this study.

### Limitations

This study has several limitations. A better design would have been preferable. Although all the parents participated voluntarily, great care had to be taken when talking with the traumatized parents. It was difficult to remind them about an extremely horrifying period in their life. Most of these parents reacted with shyness and shame when telling their stories and the interview situations were very stressful. If we had been investigating resilience in relation to war experiences, we might have got other reactions, using a longitudinal design or repeated measuring at different baselines.

The selection procedure including siblings has reduced the possibility to use advanced statistical methods. Nor were the results of the study as conclusive as could be desired. PTSD assessment is a controversial issue, especially with respect to children without a self-experienced traumatic event in their life. The idea that even *hearing about *one's parents' exposure to torture or other traumatic experiences could constitute an equivalent to a traumatic event is another limitation in the study, although the children in our study had in other ways experienced the prosecution of their parents and showed a clinical picture of PTSD or similar to PTSD. Although they showed PTSD according to DSM-IV-TR and as our assessment of Criterion A2 for PTSD was based on retrospect information from open interviews and questionnaires it may be more accurate to use the term a "PTSD-like-syndrome" when describing their situation.

Although the children spoke Swedish and were attending Swedish schools, and although A.D., the first author of the study, speaks fluently Arabic, an additional limitation is that three of the test instruments – the WISC-III, the ITIA and the SDQ – were not translated into Arabic, nor were they adjusted for use with refugee children from the Middle East. In particular, the ITIA, which is an often used Swedish instrument for measuring self-esteem in the clinical study of Swedish children and adolescents, is not an international instrument. Additional studies are needed to determine whether our use of these instruments has had a negative effect on the validity of our findings. Finally, the sample size concerning children of traumatized parents who did not develop PTSD/PTSS is very small, which reduces the generalisability of our results'.

However, despite these limitations, the study does shed light on the difficulties that these children experience and children living in other traumatized families underlines the need to find means to enhance their wellbeing and that of their parents.

## Conclusion

Children in the traumatized parents group displayed behavioral and cognitive impairments manifested mainly as PTSD-related symptoms [1]. However, not all the children in the traumatized parents group displayed such impairments; instead, they showed resilience. Most probably their resiliency was strengthened by the perception that their family was supportive despite the parents' impairments, and because they had good relations to their peers. The findings of the present study point at the importance of a supportive environment for enhancing refugee children's wellbeing. Further study is needed, however, to determine what assertive efforts could be made in the school environment and during the children's leisure-time to promote the development of resilience. These issues are essential in healthcare planning, especially in a preventive perspective.

## Authors' contributions

A.D. outlined the preliminary design of the study, performed data collection and analysis, and drafted the manuscript. B.aK. participated in the design of the study, the statistical analysis, and the presentation of results. P.-A.R. was the scientific leader of the study, participated and advised in the study design, statistical analysis, and interpretation of results. All the authors have read and approved the final manuscript.

## References

[B1] Daud A, Skoglund E, Rydelius P-A (2005). Children in families of torture victims: Transgenerational transmission of parents' traumatic experiences to their children. International Journal of Social Welfare.

[B2] Yehuda R, Teicher MH, Seckl J, Grossman RA, Moris A, Bierer LM (2007). Parental Posttraumatic Stress Disorder as a Vulnerability Factor for Low Cortisol Trait in Offspring of Holocaust Survivors. Archives of General Psychiatry.

[B3] Rydelius P-A (1981). Children of alcoholic fathers, their social adjustment and their health status over 20 years. Acta Paediatrica Scandinavica.

[B4] Rydelius P-A, Chiland C, Young JG (1994). Children of alcoholic parents: at risk to experience violence and to develop violent behaviour. Children and Violence.

[B5] Dumont M, Provost MA (1999). Resilience in Adolescents: Protective Role of Social Support, Coping Strategies, Self-Esteem, and Social Activities on Experience of Stress and Depression. Journal of Youth and Adolescence.

[B6] Otto MW, Henin A, Hirshfeld-Becker D, Pollack MH, Beiderman J, Rosenbaum JF (2007). Posttraumatic stress disorder symptoms following media Exposure to tragic events: Impact of 9/11 on children at risk for anxiety disorders. Journal of Anxiety Disorders.

[B7] Hieu MN, Thao NL (2007). Stressful Life Events, Culture, and Violence. Journal of Immigrant Health.

[B8] Garmezy N, Masten AS, Tellegen A (1984). The study of stress and competence in children: A building block for developmental psychopathology. Child Development.

[B9] Gordon KA (1995). The self-concept and motivational patterns of resilient Africa American high school students. Journal of Black Psychology.

[B10] Crawford E, Wright MO, Masten A, Roehlkepartain EC, King PE, Wagener L, Benson PL (2005). Resilience and spirituality in youth. The handbook of spiritual development in childhood and adolescence.

[B11] Garmezy N (1987). Stress, competence, and development: Continuities in the study of schizophrenic adults, children vulnerable to psychopathology, and the search for stress-resistant children. American Journal of Orthopsychiatry.

[B12] Rutter M, Pickles A, Murray R, Eaves L (2001). Testing hypotheses on specific Environmental causal effects on behavior. Psychological Bulletin.

[B13] Werner EE, Smith RS (1992). Overcoming the Odds: High Risk Children from Birth to Adulthood.

[B14] Brooks R (1994). Children at risk: Fostering resilience and hope. American Journal of Orthopsychiatry.

[B15] Sandberg S, Rutter M, Pickles A, McGuinness D, Angold A (2001). Do high-threat life events really provoke the onset of psychiatric disorder in children?. Journal of Child Psychology and Pyschiatry.

[B16] Rutter M, Giller H, Hagell A (1998). Antisocial Behavior by Young People.

[B17] Aldwin C (1994). Stress, coping and development: An integrative perspective.

[B18] Grotberg E (1999). Tapping your inner strength: How to find the resilience to deal with anything.

[B19] Kobasa SC (1979). Stressful life events, personality, and health: an inquiry into hardiness. Journal of Personality and Social Psychology.

[B20] Kliewer W, Sandier IN (1992). Locus of Control and Self-Esteem as Moderators of Stressor-Symptom Relations in Children and Adolescents. Journal of Abnormal Child Psychology.

[B21] Bartone PT (1999). Hardiness protects against war-related stress in army reserve forces. Consulting Psychology Journal: Practise and Research.

[B22] King DW, King LA, Foy W, Keane TM, Fairbank JA (1999). Post-traumatic Stress Disorder in a national sample of female and male Vietnam veterans. Risk factors, war-zone stressors, and resilience- recovery variables. Journal of Abnormal Psychology.

[B23] Ahmad A (1999). Childhood Trauma and Posttraumatic Stress Disorder. A developmental and Cross-Cultural Approach. PhD thesis No 874 A Comprehensive Summary of Uppsala Dissertations from the Faculty of Medicine.

[B24] Ahmad A, Sundelin-Wahlsten V, Sofi MA, Qahar JA, von Knorring AL (2000). Reliability and validity of a child-specific cross-cultural instrument for assessing posttraumatic stress disorder. Eur Child Adolesc Psychiatry.

[B25] Goodman R (2001). Psychometric Properties of the Strengths and Difficulties Questionnaire. Journal of the American Academy of Child and Adolescent Psychiatry.

[B26] Brigerstam P (1992). Som jag ser mig själv – en metod för studiet av äldre personers självvärdering. (As I see myself – a method for studying older people's self-assessment) Manual Gerontologiskt centrum.

[B27] Coppersmith S (1967). The antecedents of self-esteem.

[B28] Piers E (1977). The Piers-Harris children's self concept scale. Research Monograph 1.

[B29] Rosenberg M (1965). Society and the adolescent self-image.

[B30] Ouvinen-Birgerstam P (1985). Jag tycker jag är [I Think I Am].

[B31] Abd-el-Gawad G, Abrahamsson K, Hellström AL, Hjalmas K, Hanson E (2002). Health related quality of life after 5–12 years of continent Ileal Urosotomy (Kock Reservoir) in children and adolescents. Sardinian Journal of Urology and Nephrology.

[B32] Edbom T, Lichtenstein P, Granlund M, Larsson J-O (2006). Long-term relationships between symptoms of Attention-Deficit Hyperactivity Disorder and self-esteem in a prospective longitudinal study of twins. Acta Peadiatrica.

[B33] von Essen L, Enskär K, Kruger A, Larsson B, Sjöden PO (2000). Self-esteem, depression and anxiety among Swedish children and Adolescents on and off cancer treatment. Acta Peadiatrica.

[B34] American Psychiatric Association (2000). Diagnostic and statistical manual of mental Disorders (DSM-IV TR).

[B35] Cohen J (1998). Summary of Practice Parameters for the Assessment and Treatment of Children and Adolescents with Posttraumatic Stress Disorder. Journal of the American Academy of Child and Adolescent Psychiatry.

[B36] Ferren PM (1999). Comparing Perceived Self-Efficacy among Adolescent Bosnian and Croatian Refugees with and Without Posttraumatic Stress Disorder. Journal of Traumatic Stress.

[B37] af Klinteberg B, Lang S, Freidenfelt J, Alm PO, Strelau J Risk indicators and stress situations as related to pursuant disinhibitory psychosocial disturbances. Personality and extreme stress.

[B38] Pilowsky DJ, Zybert A, Valhov D (2004). Resilient Children of Injection Drug Users. Journal of the American Academy of Child and Adolescent Psychiatry.

[B39] Antonovsky A (1992). Janforum: locus of control theory. Journal of Advanced Nursing.

[B40] Antonovsky A (1996). The salutogenic model as a theory to guide health promotion. Health Promotion International.

[B41] Antonovsky A (1979). Health, stress, and coping: New perspectives on mental and physical well-being.

[B42] Antonovsky A (1987). Unraveling the mystery of health: How people manage stress and stay well.

[B43] Goldstein S, Brooks RB (2006). Handbook of Resilience in Childhood.

